# Comparison of Functional and Structural Neural Network Features in Older Adults With Depression With vs Without Apathy and Association With Response to Escitalopram: Secondary Analysis of a Nonrandomized Clinical Trial

**DOI:** 10.1001/jamanetworkopen.2022.24142

**Published:** 2022-07-27

**Authors:** Lauren E. Oberlin, Lindsay W. Victoria, Irena Ilieva, Katharine Dunlop, Matthew J. Hoptman, Jimmy Avari, George S. Alexopoulos, Faith M. Gunning

**Affiliations:** 1 Department of Psychiatry, Weill Cornell Medicine, New York, New York; 2 Institute of Geriatric Psychiatry, Weill Cornell Medicine, White Plains, New York; 3 Clinical Research Division, Nathan S. Kline Institute for Psychiatric Research, Orangeburg, New York; 4 Department of Psychiatry, NYU Grossman School of Medicine, New York, New York

## Abstract

**Importance:**

Apathy is prevalent among individuals with late-life depression and is associated with poor response to pharmacotherapy, including chronicity and disability. Elucidating brain networks associated with apathy and poor treatment outcomes can inform intervention development.

**Objectives:**

To assess the brain network features of apathy among individuals with late-life depression and identify brain network abnormalities associated with poor antidepressant response.

**Design, Setting, and Participants:**

This secondary analysis of a single-group, open-label nonrandomized clinical trial of escitalopram conducted at an outpatient geriatric psychiatry clinic enrolled 40 adults aged 59 to 85 years with major depressive disorder from July 1, 2012, to July 31, 2019.

**Interventions:**

After a 2-week washout period, participants received escitalopram titrated to a target of 20 mg/d for 12 weeks.

**Main Outcomes and Measures:**

Baseline and posttreatment magnetic resonance imaging (MRI), clinical, and cognitive assessments were conducted. Functional MRI was used to map group differences in resting state functional connectivity (rsFC) of the salience network, and diffusion MRI connectometry was performed to evaluate pathway-level disruptions in structural connectivity. The Apathy Evaluation Scale was used to quantify apathy, and the Hamilton Depression Rating Scale (HAM-D) was used to quantify the primary outcome of depression severity.

**Results:**

Forty participants (26 women [65%]; mean [SD] age, 70.0 [6.6] years [range, 59-85 years]) with depression were included; 20 participants (50%) also had apathy. Relative to nonapathetic participants with depression, those with depression and apathy had lower rsFC of salience network seeds with the dorsolateral prefrontal cortex (DLPFC), premotor cortex, midcingulate cortex, and paracentral lobule and greater rsFC with the lateral temporal cortex and temporal pole (*z* score >2.7; Bonferroni-corrected threshold of *P* < .0125). Compared with participants without apathy, those with apathy had lower structural connectivity in the splenium, cingulum, and fronto-occipital fasciculus (*t* score >2.5; false discovery rate–corrected *P* = .02). Twenty-seven participants completed escitalopram treatment; 16 (59%) achieved remission (HAM-D score <10). Lower insula-DLPFC/midcingulate cortex rsFC was associated with less symptomatic improvement (HAM-D % change) (β [*df*] = 0.588 [26]; *P* = .001) and a higher likelihood of nonremission (odds ratio, 1.041 [95% CI, 1.003-1.081]; *P* = .04) after treatment and, in regression models, was a mediator of the association between baseline apathy and persistence of depression. Lower dorsal anterior cingulate-DLPFC/paracentral rsFC was associated with residual cognitive difficulties on measures of attention (β [*df*] = 0.445 [26]; *P* = .04) and executive function (β [*df*] = 0.384 [26]; *P* = .04).

**Conclusions and Relevance:**

This study suggests that disturbances in connectivity between the salience network and other large-scale networks that support goal-directed behavior may give rise to apathy and may be associated with poor response of late-life depression to antidepressant pharmacotherapy. These network disturbances may serve as targets for novel interventions.

**Trial Registration:**

ClinicalTrials.gov Identifier: NCT01728194

## Key Points

**Question:**

What are the brain network features of apathy in late-life depression, and how are they associated with treatment outcomes?

**Findings:**

In this secondary analysis of a 12-week nonrandomized single-group trial of escitalopram titrated to 20 mg/d, compared with 20 older adults with depression without apathy, 20 older adults with depression and apathy showed distinct alterations in network connectivity among circuits that support goal-directed behavior. Apathy-related variability in functional connectivity was associated with poor antidepressant response and persistent cognitive dysfunction after escitalopram treatment.

**Meaning:**

This study suggests that, among older adults with depression, distinct network abnormalities may be associated with apathy and poor response to first-line pharmacotherapy and may serve as promising targets for novel interventions.

## Teaser

This secondary analysis of a nonrandomized clinical trial assesses the brain network features of apathy among individuals with late-life depression and identifies brain network abnormalities associated with poor antidepressant response.

## Introduction

Late-life depression (LLD) is a leading cause of disability^1^ and medical morbidity^2^ in older adulthood. One-third to one-half of patients with LLD also have apathy,^3^ a persistent and disabling syndrome of impaired motivation characterized by reduced goal-directed behavior, emotional blunting, and cognitive weaknesses.^4^ Apathy worsens clinical outcomes in LLD. Apathy results in social isolation, poor self-care, and sedentary behavior, with broad health consequences.^5,6,7,8,9,10^ Relative to older adults with depression without apathy, those with comorbid apathy are far more likely to withdraw from medical care and have a more severe clinical course, higher caregiver burden, and functional decline.^6,7^

Remission rates of LLD are especially low among those with prominent apathy.^11^ Apathy is associated with poor response of depression to selective serotonin reuptake inhibitors (SSRIs), the first-line pharmacotherapy for LLD, and efficacious nonpharmacologic treatment alternatives for this patient population are scarce.^11,12,13,14^ Despite the high prevalence and clinical impact of apathy among individuals with depression, little is known about its optimal treatment and, more broadly, about the brain-based mechanisms of apathy.

An emerging hypothesis suggests that apathy and its impact on treatment response in LLD may reflect compromise of the salience network (SN) and its large-scale network connections.^5,15,16^ The SN, which includes the insula and dorsal anterior cingulate cortex (dACC), attributes motivational value to a stimulus. The SN dynamically coordinates the activity of other large-scale networks, including the executive control network and default mode network (DMN), to guide purposeful behavior.^17,18^ The few magnetic resonance imaging (MRI) studies of apathy in depression report reduced volume in structures of the SN and preliminary evidence for disruption in functional connectivity among the SN, DMN, and executive control network.^3,5,19,20^ Abnormalities within the SN may also predispose older adults with depression to poor response to traditional pharmacotherapies.^3^ However, the network mechanisms linking apathy to poor antidepressant response in LLD are not well understood.

Identifying network abnormalities in apathy is crucial for understanding its behavioral manifestation in individuals with LLD and for developing new strategies of treatment in clinical practice. Connectometry is a novel approach to diffusion MRI analysis that quantifies the local connectome of white matter pathways.^21^ Connectometry is less susceptible to crossing fibers and partial volume effects and offers greater localization of microstructural abnormalities compared with conventional tract-based analysis^21,22,23,24,25^ but, to our knowledge, has not yet been applied to the study of apathy. Moreover, connectometry can be used alongside resting state imaging to identify network properties of apathy in major depression.

Our primary objective was to evaluate functional connectivity and structural network disturbances associated with apathy in older adults with LLD. Informed by our conceptual framework, we focused on functional connectivity of the SN and hypothesized alterations in connectivity among key nodes of the SN and other core circuits that modulate goal-directed behavior (ie, DMN, executive control network) among individuals with depression and apathy. We further applied connectometry to identify pathway-level disruptions in structural connectivity and hypothesized that compromise of frontoparietal and frontolimbic pathways would be associated with apathy in LLD. Our secondary objective was to evaluate whether apathy-related network abnormalities were associated with antidepressant response after 12 weeks of pharmacotherapy with the SSRI escitalopram. Therefore, we assessed whether pretreatment variability in connectivity associated with apathy was associated with mood and cognitive outcomes after antidepressant treatment.

## Methods

### Participants

The study, conducted at an outpatient geriatric psychiatry clinic from July 1, 2012, to July 31, 2019, included participants aged 59 to 85 years who met *Diagnostic and Statistical Manual of Mental Disorders* (Fourth Edition) (*DSM-IV*) criteria for major depressive disorder without psychotic features. Participants were recruited for a single-group, open-label escitalopram treatment trial (NCT01728194) through advertisements and clinical referrals. Participants provided written informed consent approved by the Weill Cornell Medicine and Nathan Kline Institute institutional review boards. Exclusion criteria were current or lifetime history of any Axis I psychiatric disorder other than major depressive disorder or comorbid generalized anxiety disorder, high suicide risk, history of electroconvulsive therapy, ongoing treatment with medications associated with depression (eMethods in Supplement 1), acute or severe medical illness, mild cognitive impairment or dementia, history of neurologic disease, and contraindications to undergoing MRI. The study and data are presented according to the Transparent Reporting of Evaluations With Nonrandomized Designs (TREND) reporting guideline for nonrandomized clinical trials.^26^

### Diagnostic Assessments

A *DSM-IV* diagnosis was assigned by a study clinician based on the Structured Clinical Interview for *DSM*–Revised,^27^ and inclusion required a Hamilton Depression Rating Scale (HAM-D) score of 18 or more (range, 0-76, where 0 indicates no depression and 76 indicates severe depression).^28^ Mild cognitive impairment was assessed using the Petersen criteria^29^ and determined using a previously described classification procedure.^30^ Although the primary outcome for the main clinical trial was the Montgomery-Asberg Depression Rating Scale score, several items capture core features of apathy (Montgomery-Asberg Depression Rating Scale dysphoric apathy subscale).^31,32^ Therefore, the HAM-D score was chosen as the primary depression outcome in the secondary analysis presented here.

Apathy was evaluated using the clinician-rated version of the Apathy Evaluation Scale,^33,34,35^ a psychometrically validated instrument consisting of 18 items rated on a Likert scale.^33,36,37^ Total scores range from 18 to 72, with higher scores corresponding to more severe symptoms. An examination of the psychometric properties of the Apathy Evaluation Scale found that cutoff values greater than 40.5 and 37.5^33^ had similar sensitivity (88%), although a higher cutoff value had better specificity.^34^ Accordingly, an Apathy Evaluation Scale score of more than 40.5 was considered a priori to represent clinically significant apathy.^34,35^

### Neuropsychological Assessment

A focused neuropsychological assessment conducted at baseline and after treatment included measures of processing speed, attention, memory, language, and executive functioning. This study focused on measures of attention (Digit Span Forward; Wechsler Adult Intelligence Scale, Fourth Edition) and executive function (Stroop Interference^38^ and Trail Making Test Part B − A) because they are commonly impaired among individuals with LLD and deficits often persist after antidepressant treatment.^39,40^

### Treatment Protocol

Participants previously prescribed an antidepressant underwent a 2-week washout period under the care of a study psychiatrist. Those who remained eligible after the 2 weeks were treated with escitalopram for 12 weeks: 10 mg/d for 1 week, followed by an increase to the target dose of 20 mg/d. Participants unable to tolerate 20 mg/d received either 15 mg/d or 10 mg/d, which was the minimum study dose, for the remainder of their participation. Participants completed weekly assessments of depression and side effects^41^ with trained research assistants and a research psychiatrist (eMethods in Supplement 1).

### Image Processing

Structural T1, diffusion-weighted, and T2-weighted blood oxygen level–dependent functional imaging data were acquired on a 3T Siemens Tim Trio MRI scanner. Acquisition parameters have been previously reported^30,38^ and are described along with preprocessing pipelines in the eMethods in Supplement 1.

A hypothesis-driven seed-to-whole-brain approach was used to test for group differences in resting state functional connectivity (rsFC) of the SN between participants with depression and apathy and those with depression without apathy. The left and right insula and dACC—core SN hubs—were used as a priori seed regions generated from the Human Connectome Project–derived Glasser Parcellation Atlas.^42^ Individual rsFC maps for SN seeds were generated based on correlations between the mean signal time course within each seed region and each voxel of the brain. Group-level inferential models were conducted to compare the seed-based connectivity maps of participants with and without apathy. We implemented cluster-based inference using Gaussian random field theory with a height *z* score greater than 2.3^43,44,45,46,47,48^ and a Bonferroni cluster correction of *P* < .0125 (2-tailed) to account for the number of seeds analyzed (n = 4).

Diffusion connectometry^21^ was applied to derive the correlational tractography showing an association between quantitative anisotropy and apathy.^49,50^ Connectometry measures the degree of connectivity between adjacent voxels based on the density of diffusing spins and probes group differences within subcomponents of white matter pathways.^21^ The tensor metrics were calculated, and quantitative anisotropy was extracted as the local connectome fingerprint.^51^ A *t* score threshold of 2.5 was assigned and tracked using a deterministic fiber tracking algorithm,^52^ and connectometry was performed across the whole brain, excluding the cerebellum.^53,54^ To estimate the false discovery rate (FDR), 10 000 randomized permutations were applied to the group label to obtain the null distribution of the track length. An FDR threshold of less than .05 was used to correct for multiple comparisons.^21,52^

### Statistical Analysis

The *t* test, the Mann-Whitney test, and the χ^2^ test were used to identify individual demographic and clinical variables that distinguished participants with depression without apathy from those with depression and apathy. β Weights and quantitative anisotropy values were extracted from significant clusters and fascicles that differed between groups in rsFC and connectometry analyses, respectively, to evaluate associations with clinical outcomes. Change in mood (HAM-D score) was the primary outcome, and change in attention and executive function served as secondary outcomes. Linear and logistic regression models were generated to evaluate whether group differences in baseline connectivity were associated with symptom change (percentage change in HAM-D score ) and remission of depression (HAM-D score <10),^55^ adjusting for age and baseline depression severity. Secondary linear regression models assessed the associations between baseline connectivity and change in performance (posttreatment [T_2_] − baseline [T_1_]) on measures of attention (Digit Span Forward) and executive functioning (Trail Making Test Part B-A and Stroop Interference), adjusting for age, educational level, baseline depression severity (HAM-D score), and baseline task performance.

In models with significant associations between baseline connectivity and treatment response, exploratory post hoc mediation analyses were performed^56^ to assess whether the association between presence of apathy and antidepressant response was mediated by apathy-associated abnormalities in connectivity. To estimate the indirect effect, bootstrapping (k = 5000 samples) was used to generate 95% CIs. Mediation results are considered significant if the 95% CI for the indirect path does not contain zero.^57^ Mediation models were fit in PROCESS, version 3.4.1^57^ for SPSS, version 26 (IBM SPSS).

## Results

### Sample Characteristics

Forty older adults (26 women [65%]; mean [SD] age, 70.0 [6.6] years [range, 59-85 years]) with major depressive disorder were evaluated, of whom 20 had apathy at the prespecified threshold (Apathy Evaluation Scale score >40.5). Groups did not differ significantly in age, sex, educational level, or baseline depression severity (Table 1).

**Table 1.** Demographic and Clinical Characteristics of Patients

Characteristic	Total sample (N = 40)	LLD (n = 20)	Apathy and LLD (n = 20)	*P* value
Age, mean (SD), y	70.0 (6.6)	71.3 (6.9)	68.8 (6.1)	.23
Sex, No. (%)				
Female	26 (65)	14 (70)	12 (60)	.74
Male	14 (35)	6 (30)	8 (40)	
Educational level, mean (SD), y	14.9 (2.9)	14.9 (2.4)	14.9 (3.4)	.96
AES score				
Mean (SD)	39.9 (6.7)	34.4 (3.6)	45.6 (3.4)	<.001
Range	28-53	28-40	41-53	
24-item HAM-D score, mean (SD)	24.2 (3.9)	23.2 (3.2)	25.2 (4.2)	.11

Abbreviations: AES, Apathy Evaluation Scale (clinician-rated version); HAM-D, Hamilton Depression Rating Scale; LLD, late-life depression.

### Functional Connectivity of the SN in Apathy

**Figure 1.**
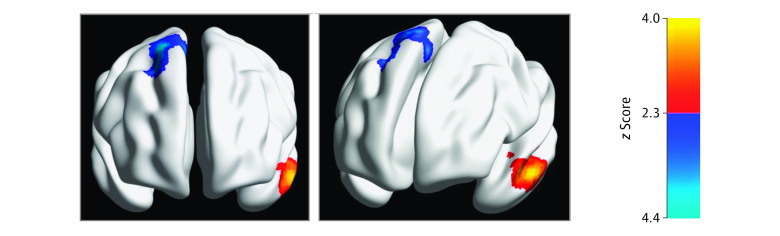
Differences in Functional Network Connectivity Associated With Apathy in Older Adults With Depression Group differences in seed-to-whole-brain functional connectivity of the left insula. Blue indicates clusters with lower connectivity with the left insula seed in participants with depression and apathy compared with participants with depression alone. Yellow indicates clusters with higher connectivity with the left insula seed in participants with apathy and depression. Cluster-based inference was implemented using Gaussian random field theory with a Bonferroni cluster correction of *P* < .0125 (2-tailed).

Compared with participants with depression alone, those with depression and apathy showed lower rsFC between the left insula seed and the right dorsolateral prefrontal cortex (DLPFC), midcingulate cortex (MCC), and premotor cortex (mean *z* score = 2.82; Bonferroni-corrected threshold of *P* < .0125) (eTable in Supplement 1). Connectivity of the left insula with the left temporal pole and middle temporal gyrus was significantly greater in participants with apathy than those without apathy (mean *z* score = 2.78; Bonferroni-corrected threshold of *P* < .0125) (Figure 1). Using the right insula seed, no significant group differences were observed.

Participants with depression and apathy showed lower rsFC between the right dACC seed and the right DLPFC, paracentral lobule (PCL), and anterior premotor cortex compared with participants with depression alone (mean *z* score = 3.0; Bonferroni-corrected threshold of *P* < .0125) (eFigure 1 in Supplement 1). Participants with apathy also showed greater rsFC between the right dACC and the left lateral temporal cortex (mean *z* score = 2.72; Bonferroni-corrected threshold of *P* < .0125). There were no group differences in connectivity using the left dACC seed.

### Structural Connectivity in Apathy

Connectometry analysis identified decreased structural connectivity (quantitative anisotropy) in the splenium of the corpus callosum, cingulum, and left inferior fronto-occipital fasciculus among participants with apathy compared with nonapathetic participants (*t* score >2.5; FDR-corrected *P* = .02) (eFigure 2 in Supplement 1). There were no areas in which quantitative anisotropy was significantly higher among those with comorbid apathy.

### Associations Between Baseline Connectivity and Antidepressant Response

**Figure 2.**
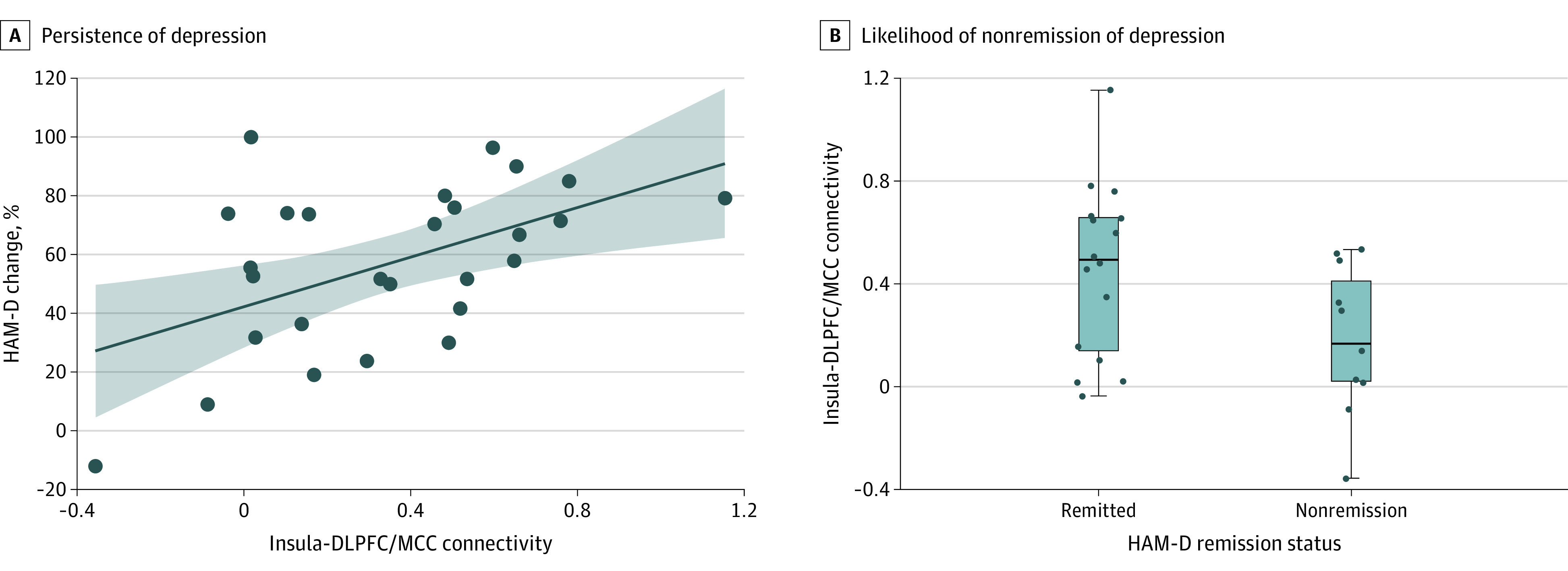
Associations Between Baseline Functional Connectivity and Antidepressant Treatment Response A, Lower pretreatment insula–dorsolateral prefrontal cortex (DLPFC)/midcingulate cortex (MCC) connectivity, which was associated with presence of apathy at baseline, was associated with greater persistence of depression (lower percentage change in Hamilton Depression Rating Scale [HAM-D] score) after treatment (β = 0.588; Δ*R*^2^ = 0.34). The *R*^2^ value indicates the change in variance that is explained (Δ*R*^2^) when functional connectivity is added to the regression model adjusted for age and baseline depression severity (HAM-D score). The shaded area indicates SE. B, Lower insula-DLPFC/MCC connectivity at baseline was associated with a higher likelihood of nonremission of depression (HAM-D score <10) after treatment (odds ratio, 1.041 [95% CI, 1.003-1.081]; *P* = .04). The box plot depicts insula-DLPFC/MCC connectivity of participants who did not achieve remission and participants who achieved remission. The median is denoted by the line within the box; 25th percentile, bottom border of box; 75th percentile, top border of box; variability outside the IQR, whiskers; and outside values, dots.

Twenty-seven participants completed the trial; 16 (59%) achieved remission (HAM-D score <10). Adjusting for age and baseline depression severity, lower insula-DLPFC/MCC connectivity at baseline was associated with less reduction in depressive symptoms (percentage change in HAM-D score) (β [*df*] = 0.588 [26]; *P* = .001) (Table 2) and a higher likelihood of nonremission (HAM-D score <10) after treatment (odds ratio, 1.041 [95% CI, 1.003-1.081]; *B* [SE] = 0.04 [0.019]; *P* = .04) (Figure 2).

**Table 2.** Parameter Estimates From Linear Regression Models Assessing Associations Between Pretreatment Salience Network Connectivity and Antidepressant Treatment Response

Parameter	Unstandardized *B* (95% CI)^a^	β Value	*P* value
**Insula-DLPFC/MCC connectivity**			
HAM-D	48.3 (21.3 to 75.2)	0.588	.001
DSF	–0.02 (–2.21 to 2.16)	−0.005	.98
Stroop Interference	4.23 (–2.6 to 11.10)	0.215	.21
Trail Making Test^b^	0.06 (–0.43 to 0.55)	0.064	.79
**dACC-DLPFC/PCL connectivity**			
HAM-D	27.01 (–13.37 to 67.38)	0.283	.18
DSF	2.7 (0.12 to 5.20)	0.445	.04
Stroop Interference	8.8 (0.52 to 17.10)	0.384	.04
Trail Making Test^b^	–0.34 (–0.90 to 0.22)	–0.330	.22

Abbreviations: dACC, dorsal anterior cingulate cortex; DLPFC, dorsolateral prefrontal cortex; DSF, Digit Span Forward; HAM-D, Hamilton Depression Rating Scale; MCC, midcingulate cortex; PCL, paracentral lobule.

^a^ HAM-D, DSF, and Stroop Interference regression model *df* = 26. The HAM-D reflects the percentage change in HAM-D score from baseline to posttreatment.

^b^ Trail Making Test regression model *df* = 21. The Trail Making Test reflects the change in difference in duration between Part A and Part B (B − A) from baseline to posttreatment.

There was no significant association between dACC connectivity and change in HAM-D score. However, there were positive associations between dACC-DLPFC/PCL connectivity and change in cognitive performance. Specifically, lower baseline dACC-DLPFC/PCL connectivity was associated with less improvement (stable or reduced performance) on measures of attention (β [*df*] = 0.445 [26]; *P* = .04) and executive function (β [*df*] = 0.384 [26]; *P* = .04) (Stroop Interference), while greater connectivity was associated with greater improvement in performance after treatment (eFigure 3 in Supplement 1). Baseline group differences in structural connectivity were not significantly associated with mood or cognitive outcomes.

Exploratory post hoc mediation analysis showed that the presence of apathy at baseline was associated with a poorer antidepressant response (lower percentage change in HAM-D score) after treatment (c path; β = *–*0.933; *P* = .02). There was an association between apathy and insula-DLPFC/MCC connectivity (a path; β = –1.20; *P* = .002), which was expected as connectivity was defined by its association with apathy in the baseline sample. There was also a significant indirect effect of insula connectivity, suggesting that group differences in insula-DLPFC/MCC connectivity mediated the association between apathy and treatment response (indirect effect β = –0.586; bootstrapped 95% CI, –1.477 to –0.072). Path coefficients indicate that baseline apathy was associated with lower insula-DLPFC/MCC connectivity which, in turn, was associated with poorer antidepressant response (see the eResults in Supplement 1 for post hoc sensitivity analyses).

## Discussion

The principal finding of this study is that, relative to participants with depression alone, those with depression and apathy showed alterations in rsFC between the SN and large-scale networks that support goal-directed behavior (DMN and executive control network) and compromised structural connectivity in a core set of brain circuits. Moreover, apathy-related alterations in rsFC were associated with reduced symptomatic improvement and a lower likelihood of remission of depression, as well as persistent cognitive dysfunction, after 12 weeks of treatment with escitalopram.

These findings support an emerging model of apathy, which proposes that apathy may arise from dysfunctional interactions among core networks (ie, SN, DMN, and executive control) that support motivated behavior.^58,59,60^ Evidence for this model has been derived largely from studies of neurodegenerative disorders and stroke,^12,14,61^ leaving the mechanisms underlying motivational disturbances among individuals with depression poorly understood.

In a sample of older adults with LLD, those with comorbid apathy showed reduced rsFC between the SN and the DLPFC, a key node of the executive control network, as well as the premotor cortex and PCL. The SN communicates with the executive control network to mobilize the cognitive resources required for goal-directed behavior^58,62^ and sends signals to primary and secondary motor cortices to facilitate action initiation.^63,64^ Functional disconnection between the SN and DLPFC may indicate reduced synchrony of systems supporting attentional control, working memory, and cognitive flexibility–core processes disrupted in apathy.^8,60,65^ Moreover, altered communication between the SN and motor systems may result in difficulties translating intention into action.^66^ These observations converge with reports in healthy adults showing associations between disturbances in rsFC between the SN and executive control network and reduced motivation,^67,68^ as well as work linking lower rsFC between the dACC and supplementary motor area to apathy among community-dwelling adults.^66^

Participants with apathy and depression also showed higher rsFC between SN seeds and the dorsal medial subsystem of the DMN. In response to salient stimuli, the SN generates control signals that downregulate the DMN in favor of task-positive networks, such as the executive control network.^17,18^ Increased rsFC with the SN may reflect difficulty modulating or disengaging the DMN, affecting the ability to generate cognitive strategies necessary for motivated behavior while ignoring irrelevant or internally directed stimuli. Consistent with this model, failure to suppress the DMN is associated with lapses in attention and decreased cognitive control performance,^69^ core features associated with apathy.^8,70^ These findings also align with a prior preliminary study showing increased rsFC between the insula and nodes of the DMN among participants with depression and apathy relative to healthy older adults.^5^

Along with functional network abnormalities, we applied diffusion connectometry to evaluate pathway-level disruptions in structural connectivity associated with apathy.^21^ Participants with comorbid apathy showed lower structural connectivity in the splenium, left inferior fronto-occipital fasciculus, and cingulum than those without apathy. The cingulum enables communication between the cingulate cortex, parietal, and medial temporal regions,^71^ and integrity of this pathway has been associated with processes disrupted in apathy,^72^ including affective processing, decision-making, and behavioral initiation.^14,60,69^ The fronto-occipital fasciculus connects frontal, insular, occipital, and parietal regions,^73,74^ and the splenium is a commissural tract that connects the posterior parietal, temporal, and occipital cortices. Efficient transmission of sensory information to the SN is needed to identify salient stimuli and guide goal-directed behavior.^17,62^ Microstructural abnormalities in these pathways may reduce interhemispheric communication and the integration of sensory signals with frontal and insular structures.^73^ Thus, along with frontolimbic fibers, compromise of pathways that facilitate the transmission of sensory signals for salience processing may be associated with the apathy trait.

More broadly, these findings collectively suggest that apathy among individuals with LLD may be associated with disturbances in SN modulation of large-scale circuits (DMN and executive control network) to facilitate goal-directed behavior. This may cause a failure of network integration,^17,18^ leading to difficulties with salience processing, action planning, and behavioral initiation that manifests clinically as apathy.

Select circuit abnormalities associated with apathy at baseline were also associated with individual differences in response to an SSRI treatment trial. Specifically, low pretreatment insula-DLPFC/MCC connectivity was associated with reduced response and remission of depression, while low dACC-DLPFC/PCL connectivity was associated with residual cognitive difficulties on measures of attention and executive function. In exploratory analyses, lower insula-DLPFC/MCC connectivity mediated the association between presence of apathy and poor antidepressant response. Thus, apathy-related variability in rsFC may predispose older adults with depression to persistent mood and cognitive deficits after traditional antidepressant pharmacotherapy. These data suggest that the effectiveness of SSRI treatment in LLD may rely on an intact ability to detect and transmit signals of salience, and highlight SN-executive control network connectivity as a potential therapeutic target for novel interventions.

### Limitations

This study has several limitations. There was no longitudinal follow-up after acute treatment and a relatively limited neuropsychological battery, so we cannot establish the persistence of treatment differences nor the specificity of cognitive associations. Although we attempted to exclude participants with mild cognitive impairment, it is possible that some participants may have had mild cognitive impairment that was not detected by our evaluation process. In addition, the study sample size is relatively small and was reduced at follow-up. As such, the longitudinal findings, including the post hoc mediation analyses, should be considered preliminary. Follow-up studies with larger samples using multiple cognitive measures and a more comprehensive diagnostic assessment of apathy^4^ will increase confidence in our results. There are also opportunities to optimize our imaging approach. The functional MRI scan was relatively short based on current standards, and a longer multiband acquisition would improve the precision of signal estimation. For cluster-based analyses, we applied a threshold commonly used in clinical trials and studies with modest sample sizes,^43,44,45,46,47,48^ although a more conservative threshold would further reduce the risk of type I error. We applied a hypothesis-driven, seed-to-whole-brain rsFC approach focused on the SN. Future studies evaluating other networks that may be disrupted in apathy, including the reward network,^16,58^ will provide further insights into its etiopathogenesis.

## Conclusions

This secondary analysis of a nonrandomized clinical trial suggests that disturbances in connectivity among a core set of brain networks that support motivated behavior (SN, DMN, and executive control network) may give rise to apathy and predispose depressed older adults to poor response to first-line pharmacotherapies. These findings contribute to our understanding of the functional neuroanatomy of motivational disturbances in depression and the network pathways linking apathy to poor clinical outcomes in LLD. Novel interventions that modulate interactions among affected circuits may help to improve clinical outcomes in this distinct subgroup of older adults with depression, for whom few effective treatments exist.

## Notes

Supplement 1.
eMethods.

eResults.
**eTable.** Coordinates (MNI) of Clusters That Differed in Functional Connectivity With Salience Network Seeds Between Participants With and Without Apathy **eFigure 1.** Differences in Dorsal Anterior Cingulate (dACC) Connectivity Associated With Apathy in Older Adults With Depression **eFigure 2.** Differences in Structural Connectivity Associated With Apathy in Older Adults With Depression **eFigure 3.** Associations Between Pretreatment Dorsal Anterior Cingulate Cortex (dACC) Connectivity and Change in Cognitive Performance Following Escitalopram Treatment
eReferences.

Supplement 2.
Data Sharing Statement

## References

[1] Ormel J, Rijsdijk FV, Sullivan M, van Sonderen E, Kempen GI. Temporal and reciprocal relationship between IADL/ADL disability and depressive symptoms in late life. J Gerontol B Psychol Sci Soc Sci. 2002;57(4):338-347. doi:10.1093/geronb/57.4.P338 12084784

[2] Katon W, Lin EHB, Kroenke K. The association of depression and anxiety with medical symptom burden in patients with chronic medical illness. Gen Hosp Psychiatry. 2007;29(2):147-155. doi:10.1016/j.genhosppsych.2006.11.005 17336664

[3] Pimontel MA, Solomonov N, Oberlin L, . Cortical thickness of the salience network and change in apathy following antidepressant treatment for late-life depression. Am J Geriatr Psychiatry. 2021;29(3):241-248. doi:10.1016/j.jagp.2020.06.007 32680763 PMC7738363

[4] Robert P, Lanctôt KL, Agüera-Ortiz L, . Is it time to revise the diagnostic criteria for apathy in brain disorders? the 2018 international consensus group. Eur Psychiatry. 2018;54:71-76. doi:10.1016/j.eurpsy.2018.07.008 30125783

[5] Yuen GS, Gunning-Dixon FM, Hoptman MJ, . The salience network in the apathy of late-life depression. Int J Geriatr Psychiatry. 2014;29(11):1116-1124. doi:10.1002/gps.4171 24990625 PMC4197060

[6] Ayers E, Shapiro M, Holtzer R, Barzilai N, Milman S, Verghese J. Symptoms of apathy independently predict incident frailty and disability in community-dwelling older adults. J Clin Psychiatry. 2017;78(5):e529-e536. doi:10.4088/JCP.15m10113 28406265 PMC5592638

[7] Yuen GS, Bhutani S, Lucas BJ, . Apathy in late-life depression: common, persistent, and disabling. Am J Geriatr Psychiatry. 2015;23(5):488-494. doi:10.1016/j.jagp.2014.06.005 25047306 PMC4277500

[8] Funes CM, Lavretsky H, Ercoli L, St Cyr N, Siddarth P. Apathy mediates cognitive difficulties in geriatric depression. Am J Geriatr Psychiatry. 2018;26(1):100-106. doi:10.1016/j.jagp.2017.06.012 28755989 PMC5725249

[9] Ismail Z, Smith EE, Geda Y, ; ISTAART Neuropsychiatric Symptoms Professional Interest Area. Neuropsychiatric symptoms as early manifestations of emergent dementia: provisional diagnostic criteria for mild behavioral impairment. Alzheimers Dement. 2016;12(2):195-202. doi:10.1016/j.jalz.2015.05.017 26096665 PMC4684483

[10] Ceïde ME, Warhit A, Ayers EI, Kennedy G, Verghese J. Apathy and the risk of predementia syndromes in community-dwelling older adults. J Gerontol B Psychol Sci Soc Sci. 2020;75(7):1443-1450. doi:10.1093/geronb/gbaa063 32374839 PMC7424283

[11] Barnhart WJ, Makela EH, Latocha MJ. SSRI-induced apathy syndrome: a clinical review. J Psychiatr Pract. 2004;10(3):196-199. doi:10.1097/00131746-200405000-00010 15330228

[12] Pimontel MA, Kanellopoulos D, Gunning FM. Neuroanatomical abnormalities in older depressed adults with apathy: a systematic review. J Geriatr Psychiatry Neurol. 2020;33(5):289-303. doi:10.1177/0891988719882100 31635522

[13] Leontjevas R, Teerenstra S, Smalbrugge M, . More insight into the concept of apathy: a multidisciplinary depression management program has different effects on depressive symptoms and apathy in nursing homes. Int Psychogeriatr. 2013;25(12):1941-1952. doi:10.1017/S1041610213001440 23992241

[14] Lanctôt KL, Agüera-Ortiz L, Brodaty H, . Apathy associated with neurocognitive disorders: recent progress and future directions. Alzheimers Dement. 2017;13(1):84-100. doi:10.1016/j.jalz.2016.05.008 27362291

[15] Gunning FM, Oberlin LE, Schier M, Victoria LW. Brain-based mechanisms of late-life depression: implications for novel interventions. Semin Cell Dev Biol. 2021;116(1):169-179. doi:10.1016/j.semcdb.2021.05.002 33992530 PMC8548387

[16] Taylor WD, Zald DH, Felger JC, . Influences of dopaminergic system dysfunction on late-life depression. Mol Psychiatry. 2022;27(1):180-191. doi:10.1038/s41380-021-01265-0 34404915 PMC8850529

[17] Uddin LQ. Salience processing and insular cortical function and dysfunction. Nat Rev Neurosci. 2015;16(1):55-61. doi:10.1038/nrn3857 25406711

[18] Sridharan D, Levitin DJ, Menon V. A critical role for the right fronto-insular cortex in switching between central-executive and default-mode networks. Proc Natl Acad Sci U S A. 2008;105(34):12569-12574. doi:10.1073/pnas.0800005105 18723676 PMC2527952

[19] Lavretsky H, Ballmaier M, Pham D, Toga A, Kumar A. Neuroanatomical characteristics of geriatric apathy and depression: a magnetic resonance imaging study. Am J Geriatr Psychiatry. 2007;15(5):386-394. doi:10.1097/JGP.0b013e3180325a16 17463189 PMC3197853

[20] Alexopoulos GS, Hoptman MJ, Yuen G, . Functional connectivity in apathy of late-life depression: a preliminary study. J Affect Disord. 2013;149(1-3):398-405. doi:10.1016/j.jad.2012.11.023 23261142 PMC3636174

[21] Yeh FC, Badre D, Verstynen T. Connectometry: a statistical approach harnessing the analytical potential of the local connectome. Neuroimage. 2016;125:162-171. doi:10.1016/j.neuroimage.2015.10.053 26499808

[22] Sihvonen AJ, Virtala P, Thiede A, Laasonen M, Kujala T. Structural white matter connectometry of reading and dyslexia. Neuroimage. 2021;241:118411. doi:10.1016/j.neuroimage.2021.118411 34293464

[23] Sobhani S, Rahmani F, Aarabi MH, Sadr AV. Exploring white matter microstructure and olfaction dysfunction in early Parkinson disease: diffusion MRI reveals new insight. Brain Imaging Behav. 2019;13(1):210-219. doi:10.1007/s11682-017-9781-0 29134611

[24] Ansari M, Rahmani F, Dolatshahi M, Pooyan A, Aarabi MH. Brain pathway differences between Parkinson’s disease patients with and without REM sleep behavior disorder. Sleep Breath. 2017;21(1):155-161. doi:10.1007/s11325-016-1435-8 27853964

[25] Sanjari Moghaddam H, Dolatshahi M, Salardini E, Aarabi MH. Association of olfaction dysfunction with brain microstructure in prodromal Parkinson disease. Neurol Sci. 2019;40(2):283-291. doi:10.1007/s10072-018-3629-2 30386933

[26] Des Jarlais DC, Lyles C, Crepaz N; TREND Group. Improving the reporting quality of nonrandomized evaluations of behavioral and public health interventions: the TREND statement. Am J Public Health. 2004;94(3):361-366. doi:10.2105/AJPH.94.3.361 14998794 PMC1448256

[27] First MB, Gibbon M. The Structured Clinical Interview for *DSM-IV* Axis I Disorders (SCID-I) and the Structured Clinical Interview for *DSM-IV* Axis II Disorders (SCID-II). In: Hilsenroth MJ, Segal DL, eds. *Comprehensive Handbook of Psychological Assessment. Vol 2: Personality Assessment*. John Wiley & Sons Inc; 2004:134-143.

[28] Williams JBW. A structured interview guide for the Hamilton Depression Rating Scale. Arch Gen Psychiatry. 1988;45(8):742-747. doi:10.1001/archpsyc.1988.01800320058007 3395203

[29] Petersen RC. Mild cognitive impairment as a diagnostic entity. J Intern Med. 2004;256(3):183-194. doi:10.1111/j.1365-2796.2004.01388.x 15324362

[30] Oberlin LE, Respino M, Victoria L, . Late-life depression accentuates cognitive weaknesses in older adults with small vessel disease. Neuropsychopharmacology. 2022;47(2):580-587. doi:10.1038/s41386-021-00973-z 33564103 PMC8674355

[31] Sockeel P, Dujardin K, Devos D, Denève C, Destée A, Defebvre L. The Lille Apathy Rating Scale (LARS), a new instrument for detecting and quantifying apathy: validation in Parkinson’s disease. J Neurol Neurosurg Psychiatry. 2006;77(5):579-584. doi:10.1136/jnnp.2005.075929 16614016 PMC2117430

[32] Parker RD, Flint EP, Bosworth HB, Pieper CF, Steffens DC. A three-factor analytic model of the MADRS in geriatric depression. Int J Geriatr Psychiatry. 2003;18(1):73-77. doi:10.1002/gps.776 12497559

[33] Marin RS, Biedrzycki RC, Firinciogullari S. Reliability and validity of the Apathy Evaluation Scale. Psychiatry Res. 1991;38(2):143-162. doi:10.1016/0165-1781(91)90040-V 1754629

[34] Clarke DE, van Reekum R, Simard M, Streiner DL, Freedman M, Conn D. Apathy in dementia: an examination of the psychometric properties of the Apathy Evaluation Scale. J Neuropsychiatry Clin Neurosci. 2007;19(1):57-64. doi:10.1176/jnp.2007.19.1.57 17308228

[35] Ishii S, Weintraub N, Mervis JR. Apathy: a common psychiatric syndrome in the elderly. J Am Med Dir Assoc. 2009;10(6):381-393. doi:10.1016/j.jamda.2009.03.007 19560715

[36] Mohammad D, Ellis C, Rau A, . Psychometric properties of apathy scales in dementia: a systematic review. J Alzheimers Dis. 2018;66(3):1065-1082. doi:10.3233/JAD-180485 30400094

[37] Clarke DE, van Reekum R, Patel J, Simard M, Gomez E, Streiner DL. An appraisal of the psychometric properties of the clinician version of the Apathy Evaluation Scale (AES-C). Int J Methods Psychiatr Res. 2007;16(2):97-110. doi:10.1002/mpr.207 17623389 PMC6878351

[38] Respino M, Hoptman MJ, Victoria LW, . Cognitive control network homogeneity and executive functions in late-life depression. Biol Psychiatry Cogn Neurosci Neuroimaging. 2020;5(2):213-221. doi:10.1016/j.bpsc.2019.10.013 31901436 PMC7010539

[39] Riddle M, Potter GG, McQuoid DR, Steffens DC, Beyer JL, Taylor WD. Longitudinal cognitive outcomes of clinical phenotypes of late-life depression. Am J Geriatr Psychiatry. 2017;25(10):1123-1134. doi:10.1016/j.jagp.2017.03.016 28479153 PMC5600662

[40] Butters MA, Young JB, Lopez O, . Pathways linking late-life depression to persistent cognitive impairment and dementia. Dialogues Clin Neurosci. 2008;10(3):345-357. doi:10.31887/DCNS.2008.10.3/mabutters 18979948 PMC2872078

[41] Lingjaerde O, Ahlfors UG, Bech P, Dencker SJ, Elgen K. The UKU Side Effect Rating Scale: a new comprehensive rating scale for psychotropic drugs and a cross-sectional study of side effects in neuroleptic-treated patients. Acta Psychiatr Scand Suppl. 1987;334(334):1-100. doi:10.1111/j.1600-0447.1987.tb10566.x 2887090

[42] Glasser MF, Coalson TS, Robinson EC, . A multi-modal parcellation of human cerebral cortex. Nature. 201611;536(7615):171-178. doi:10.1038/nature18933 27437579 PMC4990127

[43] Shokri-Kojori E, Wang GJ, Wiers CE, . β-Amyloid accumulation in the human brain after one night of sleep deprivation. Proc Natl Acad Sci U S A. 2018;115(17):4483-4488. doi:10.1073/pnas.1721694115 29632177 PMC5924922

[44] Ott CV, Macoveanu J, Bowie CR, . Change in prefrontal activity and executive functions after action-based cognitive remediation in bipolar disorder: a randomized controlled trial. Neuropsychopharmacology. 2021;46(6):1113-1121. doi:10.1038/s41386-020-00901-7 33168945 PMC8115100

[45] Saunders J, Carlson HL, Cortese F, Goodyear BG, Kirton A. Imaging functional motor connectivity in hemiparetic children with perinatal stroke. Hum Brain Mapp. 2019;40(5):1632-1642. doi:10.1002/hbm.24474 30447082 PMC6865539

[46] Gao F, Yin X, Edden RAE, . Altered hippocampal GABA and glutamate levels and uncoupling from functional connectivity in multiple sclerosis. Hippocampus. 2018;28(11):813-823. doi:10.1002/hipo.23001 30069963 PMC6251738

[47] Li R, Liao W, Yu Y, . Differential patterns of dynamic functional connectivity variability of striato-cortical circuitry in children with benign epilepsy with centrotemporal spikes. Hum Brain Mapp. 2018;39(3):1207-1217. doi:10.1002/hbm.23910 29206330 PMC6866449

[48] Lu F, Wang M, Xu S, . Decreased interhemispheric resting-state functional connectivity in male adolescents with conduct disorder. Brain Imaging Behav. 2021;15(3):1201-1210. doi:10.1007/s11682-020-00320-8 32623563

[49] Yeh FC, Wedeen VJ, Tseng WYI. Estimation of fiber orientation and spin density distribution by diffusion deconvolution. Neuroimage. 2011;55(3):1054-1062. doi:10.1016/j.neuroimage.2010.11.087 21232611

[50] Yeh FC, Wedeen VJ, Tseng WY. Generalized q-sampling imaging. IEEE Trans Med Imaging. 2010;29(9):1626-1635. doi:10.1109/TMI.2010.2045126 20304721

[51] Yeh FC, Vettel JM, Singh A, . Quantifying differences and similarities in whole-brain white matter architecture using local connectome fingerprints. PLoS Comput Biol. 2016;12(11):e1005203. doi:10.1371/journal.pcbi.1005203 27846212 PMC5112901

[52] Yeh FC, Verstynen TD, Wang Y, Fernández-Miranda JC, Tseng WYI. Deterministic diffusion fiber tracking improved by quantitative anisotropy. PLoS One. 2013;8(11):e80713. doi:10.1371/journal.pone.0080713 24348913 PMC3858183

[53] Yeh FC, Panesar S, Barrios J, . Automatic removal of false connections in diffusion MRI tractography using topology-informed pruning (TIP). Neurotherapeutics. 2019;16(1):52-58. doi:10.1007/s13311-018-0663-y 30218214 PMC6361061

[54] Yeh FC, Liu L, Hitchens TK, Wu YL. Mapping immune cell infiltration using restricted diffusion MRI. Magn Reson Med. 2017;77(2):603-612. doi:10.1002/mrm.26143 26843524 PMC8052951

[55] Kyle PR, Lemming OM, Timmerby N, Søndergaard S, Andreasson K, Bech P. The validity of the different versions of the Hamilton Depression Scale in separating remission rates of placebo and antidepressants in clinical trials of major depression. J Clin Psychopharmacol. 2016;36(5):453-456. doi:10.1097/JCP.0000000000000557 27525966

[56] Kraemer HC, Wilson GT, Fairburn CG, Agras WS. Mediators and moderators of treatment effects in randomized clinical trials. Arch Gen Psychiatry. 2002;59(10):877-883. doi:10.1001/archpsyc.59.10.877 12365874

[57] Hayes AF. Introduction to Mediation, Moderation, and Conditional Process Analysis: A Regression-Based Approach. 2nd ed. Guilford Publications; 2018.

[58] Husain M, Roiser JP. Neuroscience of apathy and anhedonia: a transdiagnostic approach. Nat Rev Neurosci. 2018;19(8):470-484. doi:10.1038/s41583-018-0029-9 29946157

[59] Tay J, Lisiecka-Ford DM, Hollocks MJ, . Network neuroscience of apathy in cerebrovascular disease. Prog Neurobiol. 2020;188:101785. doi:10.1016/j.pneurobio.2020.101785 32151533

[60] Le Heron C, Apps MAJ, Husain M. The anatomy of apathy: a neurocognitive framework for amotivated behaviour. Neuropsychologia. 2018;118(pt B):54-67. doi:10.1016/j.neuropsychologia.2017.07.003 28689673 PMC6200857

[61] Raimo S, Santangelo G, D’Iorio A, Trojano L, Grossi D. Neural correlates of apathy in patients with neurodegenerative disorders: an activation likelihood estimation (ALE) meta-analysis. Brain Imaging Behav. 2019;13(6):1815-1834. doi:10.1007/s11682-018-9959-0 30238208

[62] Uddin LQ, Nomi JS, Hébert-Seropian B, Ghaziri J, Boucher O. Structure and function of the human insula. J Clin Neurophysiol. 2017;34(4):300-306. doi:10.1097/WNP.0000000000000377 28644199 PMC6032992

[63] Kouneiher F, Charron S, Koechlin E. Motivation and cognitive control in the human prefrontal cortex. Nature Neurosci. 2009;12(7):939-945. doi:10.1038/nn.2321 19503087

[64] Chong TTJ, Apps M, Giehl K, Sillence A, Grima LL, Husain M. Neurocomputational mechanisms underlying subjective valuation of effort costs. PLoS Biol. 2017;15(2):e1002598. doi:10.1371/journal.pbio.1002598 28234892 PMC5325181

[65] Saleh Y, Le Heron C, Petitet P, . Apathy in small vessel cerebrovascular disease is associated with deficits in effort-based decision making. Brain. 2021;144(4):1247-1262. doi:10.1093/brain/awab013 33734344 PMC8240747

[66] Bonnelle V, Manohar S, Behrens T, Husain M. Individual differences in premotor brain systems underlie behavioral apathy. Cereb Cortex. 2016;26(2):807-819. doi:10.1093/cercor/bhv247 26564255 PMC4712805

[67] Huskey R, Craighead B, Miller MB, Weber R. Does intrinsic reward motivate cognitive control? a naturalistic-fMRI study based on the synchronization theory of flow. Cogn Affect Behav Neurosci. 2018;18(5):902-924. doi:10.3758/s13415-018-0612-6 29923098

[68] Morris LS, Kundu P, Dowell N, . Fronto-striatal organization: defining functional and microstructural substrates of behavioural flexibility. Cortex. 2016;74:118-133. doi:10.1016/j.cortex.2015.11.004 26673945 PMC4729321

[69] Le Heron C, Holroyd CB, Salamone J, Husain M. Brain mechanisms underlying apathy. J Neurol Neurosurg Psychiatry. 2019;90(3):302-312. doi:10.1136/jnnp-2018-318265 30366958 PMC6518466

[70] Montoya-Murillo G, Ibarretxe-Bilbao N, Peña J, Ojeda N. The impact of apathy on cognitive performance in the elderly. Int J Geriatr Psychiatry. 2019;34(5):657-665. doi:10.1002/gps.5062 30672026 PMC6594084

[71] Bubb EJ, Metzler-Baddeley C, Aggleton JP. The cingulum bundle: anatomy, function, and dysfunction. Neurosci Biobehav Rev. 2018;92:104-127. doi:10.1016/j.neubiorev.2018.05.008 29753752 PMC6090091

[72] Beckmann M, Johansen-Berg H, Rushworth MFS. Connectivity-based parcellation of human cingulate cortex and its relation to functional specialization. J Neurosci. 2009;29(4):1175-1190. doi:10.1523/JNEUROSCI.3328-08.2009 19176826 PMC6665147

[73] Wu Y, Sun D, Wang Y, Wang Y. Subcomponents and connectivity of the inferior fronto-occipital fasciculus revealed by diffusion spectrum imaging fiber tracking. Front Neuroanat. 2016;10(SEP):88. doi:10.3389/fnana.2016.00088 27721745 PMC5033953

[74] Nomi JS, Schettini E, Broce I, Dick AS, Uddin LQ. Structural connections of functionally defined human insular subdivisions. Cereb Cortex. 2018;28(10):3445-3456. doi:10.1093/cercor/bhx211 28968768 PMC6132280

