# Supplementary material for: Comparison of Functional and Structural Neural Network Features in Older Adults With Depression With vs Without Apathy and Association With Response to Escitalopram: Secondary Analysis of a Nonrandomized Clinical Trial
Source: JAMA Netw Open. 2022 Jul 27;5(7):e2224142. doi: 10.1001/jamanetworkopen.2022.24142 (PMC9331093; doi:10.1001/jamanetworkopen.2022.24142)
Supplement: Supplement 1. — eMethods. eResults. eTable. Coordinates (MNI) of Clusters That Differed in Functional Connectivity With Salience Network Seeds Between Participants With and Without Apathy eFigure 1. Differences in Dorsal Anterior Cingulate (dACC) Connectivity Associated With Apathy in Older Adults With Depression eFigure 2. Differences in Structural Connectivity Associated With Apathy in Older Adults With Depression eFigure 3. Associations Between Pretreatment Dorsal Anterior Cingulate Cortex (dACC) Connectivity and Change in Cognitive Performance Following Escitalopram Treatment eReferences. [file jamanetwopen-e2224142-s001.pdf]

Supplementary Online Content

Oberlin LE, Victoria LW, Ilieva I, et al. Comparison of functional and structural neural network features in older adults with depression with vs without apathy and association with response to escitalopram: secondary analysis of a nonrandomized clinical trial. *JAMA Netw Open.* 2022;5(7):e2224142. doi:10.1001/jamanetworkopen.2022.24142

eMethods.
eResults.
eTable. Coordinates (MNI) of Clusters That Differed in Functional Connectivity With Salience Network Seeds Between Participants With and Without Apathy
eFigure 1. Differences in Dorsal Anterior Cingulate (dACC) Connectivity Associated With Apathy in Older Adults With Depression
eFigure 2. Differences in Structural Connectivity Associated With Apathy in Older Adults With Depression
eFigure 3. Associations Between Pretreatment Dorsal Anterior Cingulate Cortex (dACC) Connectivity and Change in Cognitive Performance Following Escitalopram Treatment
eReferences.

This supplementary material has been provided by the authors to give readers additional information about their work.

eMethods.

Participants and Assessments

Exclusion criteria were assessed by study clinicians through clinical interview. History of psychotherapy was not exclusionary, but current participation in psychotherapy was an exclusionary criterion. Patients taking fluoxetine were not considered for the study because of the longer washout period (> 2 weeks) required. Individuals receiving ongoing treatment with medications associated with depression (e.g. steroids, clonidine, tamoxifen, cimetidine) were not considered for inclusion.

Study procedures including assessments and weekly evaluations of depression and side effects were conducted at the Weill Cornell Institute of Geriatric Psychiatry, an outpatient geriatric psychiatry clinic. Neuroimaging data was collected at the Nathan Kline Institute. Study enrollment occurred from July 2012 to July 2019. Clinical and cognitive data were obtained during the baseline visit, which followed the two-week washout period. Posttreatment clinical and cognitive data were collected during the final (Week 12) evaluation. For neuropsychological outcome variables, higher values indicate improvement in task performance from baseline (T_1) to posttreatment (T_2).

Treatment Protocol

Participants completed weekly assessments which included administration of mood (Hamilton Depression Rating Scale; HAM-D) and side effect rating scales by trained research assistants and a brief evaluation by a research psychiatrist. Medication side effects were assessed weekly using the UKU Side Effect Rating Scale[1].

MRI Data Acquisition

Imaging data were acquired on a 3T Siemens Tim Trio equipped with a 32-channel head coil at the Nathan Kline Institute for Psychiatric Research. Anatomical imaging included a turbo dual echo scan, with high-resolution whole brain images acquired using a 3D T1-weighted MPRAGE. The acquisition parameters for the T1-weighted MPRAGE were as follows: TR = 2500 ms, TE = 3.5 ms, slice thickness = 1 mm, TI= 1200 ms, 192 axial slices, matrix = 256×256 (voxel size = 1 mm isovoxel), FOV= 256 mm, IPAT = 2, flip angle = 8°.

Diffusion weighted imaging data of 72 contiguous slices (parallel to the AC-PC plane) were acquired with a spin-echo EPI pulse sequence using 30 diffusion-encoding directions (b-value = 1000 s/mm^2) with 6 nondiffusion weighted b=0 s/mm^2 and the following parameters: TR = 9000 ms, TE = 91 ms, FOV = 256×256 mm, flip angle = 90°, voxel size = 2 × 2 × 2 mm^3, acquisition matrix size = 128×128.

Resting state images were acquired using a single-shot, T2-weighted echo planar blood oxygen level–dependent contrast image, which allowed whole brain coverage (TR = 2500 ms, TE = 30 ms, flip angle = 80°, slice thickness = 3 mm, 38 axial slices, matrix = 72 x 72, 3-mm isovoxel, FOV = 216 mm, integrated parallel acquisition techniques factor = 2). Acquisition time was 6 minutes, 15 seconds (150 volumes). Patients were instructed to stay awake with eyes closed and wakefulness was verified at the end of the sequence by the MR technician.

fMRI Preprocessing

MRI data were preprocessed using the FMRIB Software Library (FSL) and the Analysis of Functional Neuroimages (AFNI) software. High resolution T1 anatomical images were registered to standard space using 1) an affine transformation to align anatomic images to the anterior and posterior commissures of the standard Montreal Neurologic Institute (MNI)-152 template, and 2) a nonlinear transformation to warp the T1 image to the same template. Rigid-body motion correction was performed, followed by affine co-registration of motion-corrected resting state images to the anterior-posterior-aligned T1 anatomic image. FSL's FNIRT tool was used for nonlinear transformation to the MNI-152 template. Preprocessing of normalized functional images included brain extraction, temporal filtering, and spatial smoothing with a 6mm full-width half-maximum Gaussian kernel. Additional correction for motion and physiological noise components was executed using ICA-AROMA, which uses an independent component analysis-based strategy to automatically regress out artifacts related to motion and physiological sources. A final denoising step was conducted in AFNI and

© 2022 Oberlin LE et al. *JAMA Network Open.*

modeled the following: linear trends, 12 variables corresponding to the demeaned and first temporal derivatives of the translational and directional motion traces outputted from rigid-body motion correction, fast ANATICOR regression to account for local hardware and respiratory artifacts, and the first three principal components isolated from a lateral ventricle mask, and temporal bandpass filtering (0.01—0.1 Hz). The residual BOLD time course was retained for functional connectivity analysis. To assess scan quality following preprocessing and denoising, framewise displacement[2], whole-brain global correlation (GCOR)[3], and whole-brain mean tSNR were extracted for each scan. If the scan was an outlier on two of the three aforementioned measures (defined as 1.5 times the 25th and 75th percentile for each metric[4]), the scan was considered for removal. The mean framewise displacement was 0.22 mm (interquartile range = 0.21) and we did not remove any participants from analysis due to motion parameters.

Diffusion Data Preprocessing and Analysis

Diffusion data was processed using tools in FSL and DSI-Studio. Each participant's data was eddy current corrected by affine registration to the first b=0 image, followed by the removal of non-brain tissue using the Brain Extraction Tool (BET). Next, DTIfit was used to calculate the diffusion tensor at each voxel.

This data was fed into DSI-Studio, and diffusion data was reconstructed in the MNI space using q-space diffeomorphic reconstruction[5] to obtain the spin distribution function[6]. A diffusion sampling length ratio of 1.25 was used. The output resolution in diffeomorphic reconstruction was 2 mm isotropic. The restricted diffusion was quantified using restricted diffusion imaging[7]. The tensor metrics were calculated, and the quantitative anisotropy (QA) was extracted as the local connectome fingerprint[8] and used in the connectometry analysis[9]. A T-score threshold of 2.5 was assigned and tracked using a deterministic fiber tracking algorithm[10] to obtain correlational tractography of commissural and association tracts. Connectometry was performed across the whole brain (excluding cerebellum) and the tracks were filtered by topology-informed pruning with 10 iterations[11].

© 2022 Oberlin LE et al. *JAMA Network Open.*

eResults.

Associations Between Baseline Connectivity and Antidepressant Response

To assess the specificity of the indirect relationship between apathy and antidepressant response through insula rsFC in the exploratory *post hoc* mediation analysis, we substituted connectivity of the dACC as the mediator and the indirect effect was not significant. In a separate *post hoc* sensitivity analysis, a continuous measure of apathy severity (AES) served as the mediator and insula-DLPFC/MCC connectivity as the predictor. In this model, the indirect effect was non-significant, providing additional support for insula-DLPFC/MCC connectivity as an indirect path linking presence of apathy to antidepressant treatment response. In two secondary exploratory models, we tested whether connectivity of the dACC mediated the associations between apathy and change in cognition, and no significant mediation effects were observed.

eTable. Coordinates (MNI) of Clusters That Differed in Functional Connectivity With Salience Network Seeds Between Participants With and Without Apathy

	MNI Coordinates						Z statistic
	x		y		z		Mean (Max)
**Left insula seed**							
**Cluster 1**							2.82 (4.46)
Right DLPFC	23		19		57		
Right midcingulate cortex	2		-8		54		
Right premotor cortex	22		-11		68		
**Cluster 2**							2.78 (4.10)
Left temporal pole	-54		1		-15		
Left middle temporal gyrus	-57		-11		-15		
**Right dACC seed**							
**Cluster 1**							3.0 (5.37)
Right DLPFC	28		7		59		
Right paracentral lobule	25		10		59		
Right premotor cortex	26		7		52		
**Cluster 2**							2.72 (3.64)
Left lateral temporal cortex	-45		19		-32		

Cluster-based inference was implemented using Gaussian Random Field Theory with a height z-score > 2.3 and a Bonferroni cluster correction of $p < 0.0125$ (two-tailed). Abbreviations: dACC = dorsal anterior cingulate cortex; DLPFC = dorsolateral prefrontal cortex.

© 2022 Oberlin LE et al. *JAMA Network Open.*

eFigure 1. Differences in Dorsal Anterior Cingulate (dACC) Connectivity Associated With Apathy in Older Adults With Depression

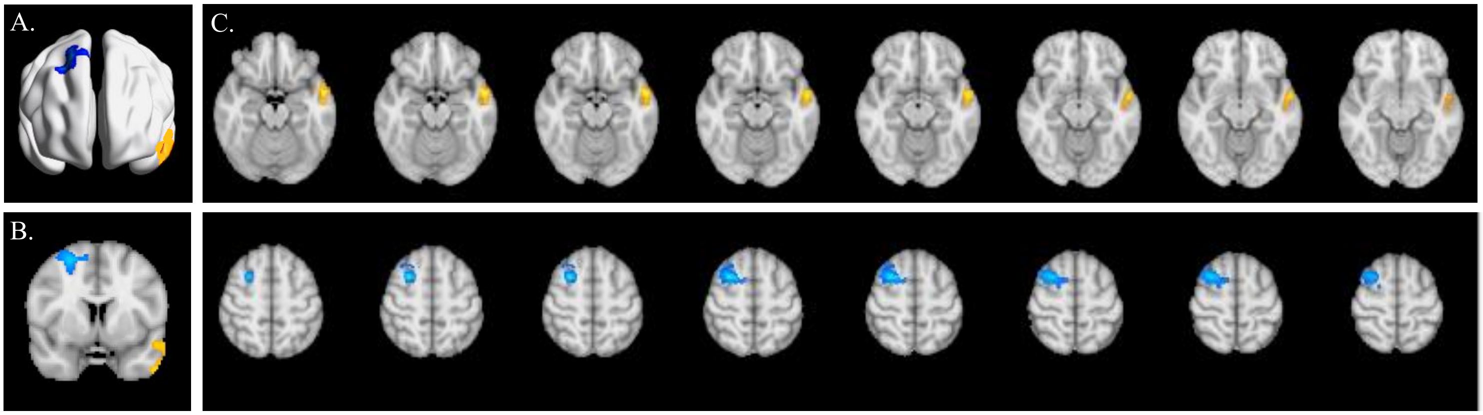

Group differences in seed-to-whole brain functional connectivity of the right dorsal anterior cingulate cortex (dACC) *Blue*: Clusters with lower connectivity with the right dACC seed in participants with depression and apathy compared to participants with depression without apathy. *Yellow*: Clusters with higher connectivity with the right dACC seed in participants with apathy and depression. A) 3-dimensional rendering of significant clusters. B) Coronal view, coordinates (MNI): x = 18, y = 7, z = 58. C) Depiction of clusters across multiple axial slices. Cluster-based inference was implemented using Gaussian Random Field Theory with a Bonferroni cluster correction of $p < 0.0125$ (two-tailed).

© 2022 Oberlin LE et al. *JAMA Network Open.*

eFigure 2. Differences in Structural Connectivity Associated With Apathy in Older Adults With Depression

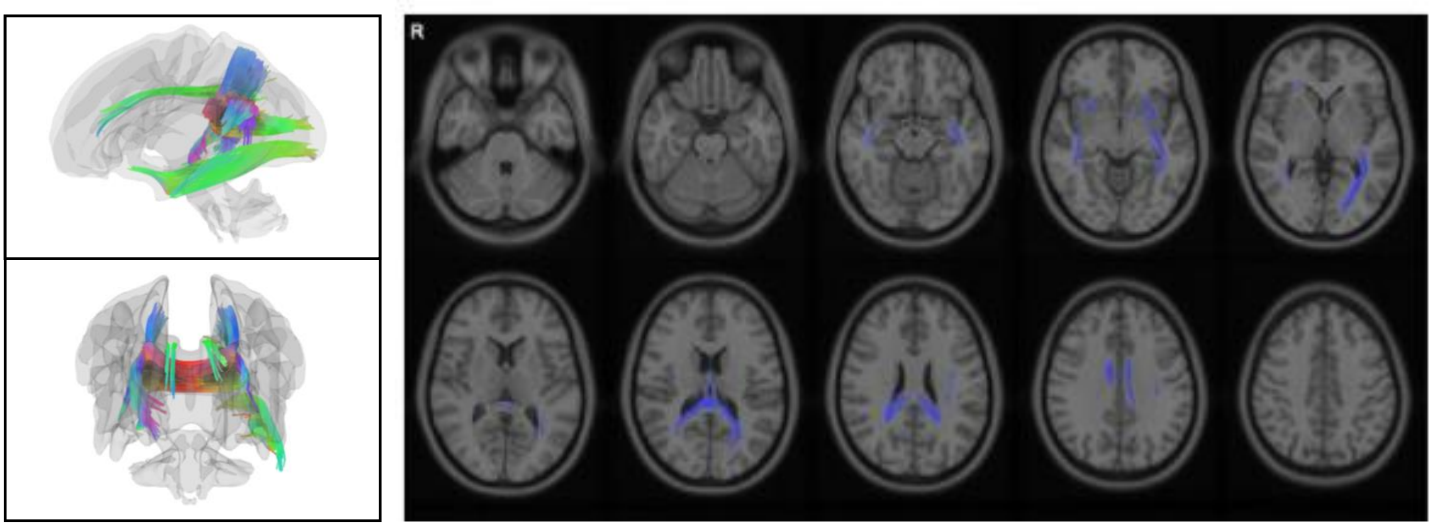

Fascicles revealed by diffusion connectometry to have lower structural connectivity (quantitative anisotropy) among participants with depression and apathy when compared to nonapathetic participants with depression, including the splenium of the corpus callosum, cingulum, and left inferior fronto-occipital fasciculus (false discovery rate [FDR]-corrected P = 0.02). Streamlines smoothed for visualization.

© 2022 Oberlin LE et al. *JAMA Network Open.*

eFigure 3. Associations Between Pretreatment Dorsal Anterior Cingulate Cortex (dACC) Connectivity and Change in Cognitive Performance Following Escitalopram Treatment

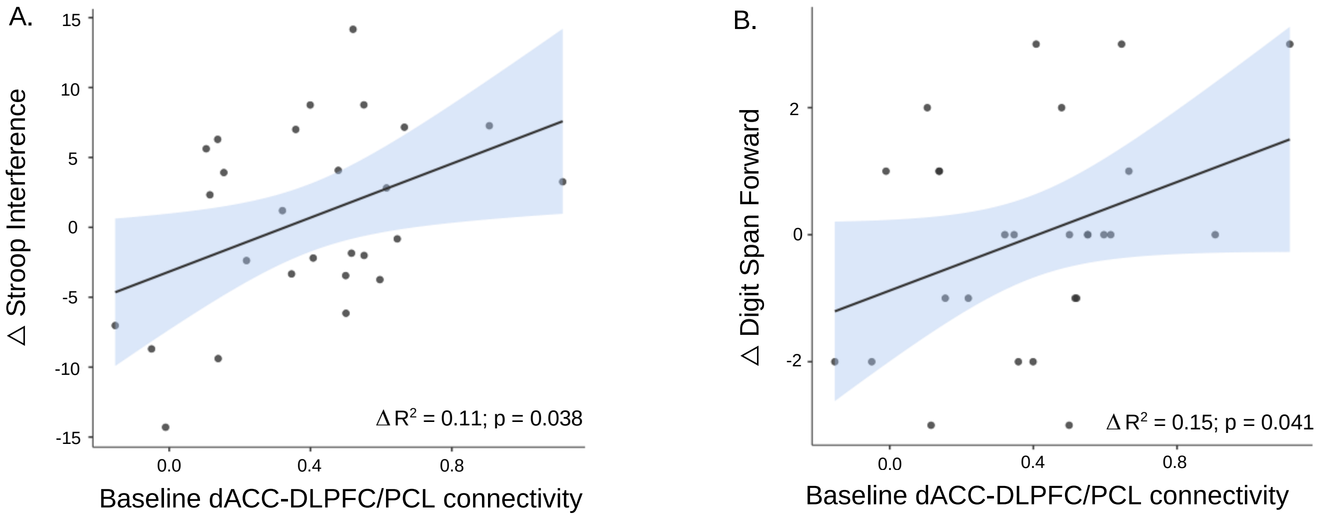

Lower dorsal anterior cingulate-dorsolateral prefrontal cortex/paracentral lobule (dACC-DLPFC/PCL) connectivity at baseline was associated with less improvement (stable or reduced performance) on measures of executive function (A; Stroop Interference) and attention (B; Digit Span Forward) following 12 weeks of escitalopram treatment. R^2 values reflect change in variance explained (ΔR^2) when adding functional connectivity as a predictor in regression models adjusting for age, education, baseline depression severity (HAM-D), and baseline task performance. Shaded areas reflect standard errors.

© 2022 Oberlin LE et al. *JAMA Network Open.*

eReferences.

1. Lingjærde O, Ahlfors UG, Bech P, Dencker SJ, Elgen K. The UKU side effect rating scale: A new comprehensive rating scale for psychotropic drugs and a cross-sectional study of side effects in neuroleptic-treated patients. *Acta Psychiatr Scand*. 1987;76(334):1-100. doi:10.1111/j.1600-0447.1987.tb10566.

2. Power JD, Mitra A, Laumann TO, Snyder AZ, Schlaggar BL, Petersen SE. Methods to detect, characterize, and remove motion artifact in resting state fMRI. *Neuroimage*. 2014;84:320–341.

3. Saad ZS, Reynolds RC, Jo HJ, Gotts SJ, Chen G, Martin A, et al. Correcting brain-wide correlation differences in resting-state FMRI. *Brain Connect*. 2013;3:339–352.

4. Dunlop, K., Victoria, L. W., Downar, J., Gunning, F. M., & Liston, C. Accelerated brain aging predicts impulsivity and symptom severity in depression. *Neuropsychopharmacology*. 2021;46(5), 911-919.

5. Yeh F, Wedeen V, Tseng Y. Generalized q-Sampling Imaging. *IEEE Trans Med Imaging*. 2010;29(9):1626-1635. Accessed October 21, 2021.

6. Yeh FC, Wedeen VJ, Tseng WYI. Estimation of fiber orientation and spin density distribution by diffusion deconvolution. *Neuroimage*. 2011;55(3):1054-1062. doi:10.1016/j.neuroimage.2010.11.087

7. Yeh FC, Liu L, Hitchens TK, Wu YL. Mapping immune cell infiltration using restricted diffusion MRI. *Magn Reson Med*. 2017;77(2):603-612. doi:10.1002/mrm.26143

8. Yeh FC, Vettel JM, Singh A, et al. Quantifying Differences and Similarities in Whole-Brain White Matter Architecture Using Local Connectome Fingerprints. *PLoS Comput Biol*. 2016;12(11). doi:10.1371/JOURNAL.PCBI.1005203

9. Yeh FC, Badre D, Verstynen T. Connectometry: A statistical approach harnessing the analytical potential of the local connectome. *Neuroimage*. 2016;125:162-171. doi:10.1016/j.neuroimage.2015.10.053

10. Yeh FC, Verstynen TD, Wang Y, Fernández-Miranda JC, Tseng WYI. Deterministic diffusion fiber tracking improved by quantitative anisotropy. *PLoS One*. 2013;8(11). doi:10.1371/JOURNAL.PONE.0080713

11. Yeh FC, Panesar S, Barrios J, et al. Automatic Removal of False Connections in Diffusion MRI Tractography Using Topology-Informed Pruning (TIP). *Neurotherapeutics*. 2019;16(1):52-58. doi:10.1007/S13311-018-0663-Y

© 2022 Oberlin LE et al. *JAMA Network Open.*
