# Supplementary material for: Comparison of Functional and Structural Neural Network Features in Older Adults With Depression With vs Without Apathy and Association With Response to Escitalopram: Secondary Analysis of a Nonrandomized Clinical Trial
Source: JAMA Netw Open. 2022 Jul 27;5(7):e2224142. doi: 10.1001/jamanetworkopen.2022.24142 (PMC9331093; doi:10.1001/jamanetworkopen.2022.24142)
Supplement: Supplement 2. — Data Sharing Statement [file jamanetwopen-e2224142-s002.pdf]

Data Sharing Statement

Oberlin. Comparison of Functional and Structural Neural Network Features in Older Adults With Depression With vs Without Apathy and Association With Response to Escitalopram. *JAMA Netw Open*. Published July 27, 2022. doi:10.1001/jamanetworkopen.2022.24142

Data
Data available: Yes
Data types: Deidentified participant data, Data dictionary
How to access data: Request for data to corresponding author, Dr. Faith Gunning (fgd2002@med.cornell.edu).
When available: With publication

Supporting Documents
Document types: None

Additional Information
Who can access the data: Researchers requesting data whose proposed use of data has been approved.
Types of analyses: For a specified purpose.
Mechanisms of data availability: With investigator support, after approval of a proposal.
